# Knowledge on Obstetric Danger Signs During Pregnancy and Associated Factors Among Married Men in Chencha Town, Southern Ethiopia Regional State, 2022: A Community-Based Cross-Sectional Study

**DOI:** 10.1155/jp/8311265

**Published:** 2025-10-02

**Authors:** Aster Dure, Nega Degefu, Kinde Kibe, Addisalem Haile, Eden Sileshi, Arega Abebe, Amanuel Elias, Marishet Mekonen

**Affiliations:** ^1^School of Nursing, College of Medicine and Health Sciences, Arba Minch University, Arba Minch, Ethiopia; ^2^Department of Midwifery, College of Medicine and Health Sciences, Wachemo University, Durame Campus, Durame, Ethiopia

**Keywords:** danger signs during pregnancy, Ethiopia, husbands' knowledge

## Abstract

**Background:**

Poor knowledge of danger signs during pregnancy can have serious consequences on the health of both the mother and the baby. In addition to this inability to recognize signs of obstetric complications, it serves as a barrier to making a decision to access healthcare and therefore is one of the factors responsible for the first level of delay that contributes to maternal mortality. Previous studies conducted in the country were focused on assessing maternal knowledge about obstetric danger signs during pregnancy. However, there is a scarcity of information or little is known about the current knowledge of the husbands about obstetric danger signs during pregnancy and influencing factors regarding the obstetric danger signs during pregnancy in Ethiopia, particularly in Chencha town, southern regional state.

**Methods:**

A community-based cross-sectional study was conducted among 422 husbands living in selected kebeles in Chencha town from September 1 to 30, 2022. A random sampling technique was used to select kebeles in Chencha town, southern region. Data were collected using a structured and pretested questionnaire. Collected data were analyzed using the statistical package for social science (SPSS) Version 25. Results were presented in the form of percentage, frequency tables, and pie charts. Binary logistic regression was performed to check for an association between independent and outcome variables at *p* < 0.05 and a 95% confidence interval (CI). Then, the variables with *p* value < 0.25 were entered into multivariate logistic regression to identify statistically significant variables. Before adjusting in the multivariable analysis, the candidate variables for the multivariable analysis were checked for multicollinearity using the variance inflation factor, which ranged from 1.1 to 1.87. The Hosmer–Lemeshow test was used to assess the model's fitness (0.124).

**Results:**

The study found that 45.5% (95% CI: 41%–50%) of husbands are aware of the danger sign during pregnancy. Factors such as the wife's secondary educational level (AOR = 4.700, 95% CI: 2.330–9.478), more than secondary educational level (AOR = 3.132, 95% CI: 1.549–6.364), previous obstetric complications (AOR = 1.796, 95% CI: 1.145–2.817), access to media information (AOR = 1.881, 95% CI: 1.117–3.166), and follow-up of antenatal care (AOR = 1.839, 95% CI: 1.175–2.880) were statistically significantly associated with married men's knowledge of obstetric danger signs during pregnancy.

**Conclusions:**

This study indicated that the current knowledge of husbands about the danger sign during pregnancy was low. Therefore, strengthening the provision of information on danger signs during pregnancy in ANC and information on behavioral communication of husbands regarding partner support is recommended.

## 1. Introduction

Pregnancy-related life-threatening disorders are referred to as “danger signs during pregnancy.” Prenatal hemorrhage, hyperemesis, eclampsia, vaginal bleeding, hypertension, severe headache, hand and facial edema, gush blood from the vagina, and convulsions are the most common warning symptoms that can increase the chance of maternal mortality [1–3].

Lack of knowledge of pregnancy danger signs has a burden on maternal mortality [4]. The World Health Organization (WHO) estimates that every day in 2020, approximately 800 women would die from preventable causes during pregnancy and childbirth. In 2020, there was a maternal death approximately every 2 min. Maternal mortality ratios, or the number of maternal deaths per 100,000 live births, decreased by almost 34% globally between 2000 and 2020. Approximately 95% of all maternal deaths occurred in low-income and lower middle-income nations in 2020 [5].

The most common causes of maternal mortality in Ethiopia are sepsis, hemorrhage, pregnancy-related hypertension, and abortion. These problems can be prevented with early detection and expert institutional care. By 2020, MMR should drop to 199/100,000 live births, according to plans of the Ethiopian Federal Ministry of Health [6]. According to the global target of SDGs for maternal health, by 2030, the MMR (maternal mortality ratio) should be lowered to less than 70 per 100,000 live births globally, and no nation should have a national MMR of more than 140 per 100,000 live births [7].

The consequences of a lack of awareness of danger signs cause partners to delay seeking obstetric care, increasing maternal mortality and morbidity rates globally. That is why one of the main causes of maternal death is delayed medical attention, which may be related to ignorance of obstetric warning signs. In addition to this, research has shown that knowledge of danger signs in pregnancy by pregnant individuals themselves is low, and it results in a reduced tendency to seek skilled attendance at birth and referral in case of complications. It is important that we place more emphasis on husbands about these issues; this is because male partners or husbands make the majority of decisions regarding many other matters in the home, including those regarding women's and children's health rely on them [8–10].

The WHO created maternal and newborn health counseling to address the aforementioned issue by educating women and their families about the early detection and recognition of danger signs during pregnancy and complications as part of birth and emergency planning [11]. An interventional study in Ethiopia that evaluated a community-based intervention package to increase awareness of obstetric warning signs reveals that the intervention increased the proportion from 48.6% at the beginning to 62.3% at the end [12]. The need to “promote gender equality in all spheres of life and to promote men to take responsibility for their sexual and reproductive behavior and social and family roles” has been highlighted by the International Conference on Population and Development (ICPD) [13].

The knowledge of husbands regarding pregnancy danger signs is low in low- and middle-income countries, despite the fact that ignorance of these signs increases maternal mortality [8, 14, 15]. A study conducted in Dessie town, Ethiopia, found that the prevalence of husband knowledge about pregnancy danger signs was 49.3% [15].

According to available evidence, socioeconomic status, educational status, number of children, occupation, monthly income, and place of delivery were factors that affect knowledge of husbands about danger signs during pregnancy [15, 16].

Previous studies conducted in the country were focused on assessing maternal knowledge about danger signs during pregnancy. Other studies assessed husband knowledge about danger signs during pregnancy before the occurrence of coronavirus disease 2019 (COVID-19), which has its own negative impact on maternal and child health. However, there is a scarcity of information, or little is known about the current knowledge of husbands about danger signs during pregnancy and influencing factors regarding the danger signs during pregnancy in Ethiopia.

Therefore, the objective of the current study was to evaluate husbands' knowledge of pregnancy danger signs during pregnancy and associated factors in Gamo Zone in southern Ethiopia.

## 2. Methods and Materials

### 2.1. Study Area, Period, and Design

A community-based cross-sectional study was conducted among husbands of women who gave birth in the last 12 months. The study was carried out in Chencha town, Gamo Zone, South Ethiopia, from April 1 to 10, 2022. The town of Chencha is located 530 km southwest of Addis Ababa, the capital of Ethiopia, and 298 km from the regional town of Hawassa. The town has a total of eight kebeles and is situated at an altitude of 2732 m above sea level. The town has a total population of 32,288, and among these, 16,466 were women, and 15,822 were men. The total number of husbands of women who gave birth during the last 12 months in Chencha town is 1116. The town has one district hospital, one health center, three private clinics, and eight health posts.

### 2.2. Study Population and Eligibility Criteria

The source population was all the husbands of women who gave birth during the last 12 months in eight kebeles of Chencha town. The study population was sampled from husbands of women who gave birth in the last 12 months in five selected kebeles in Chencha city that met the inclusion criteria.

Husbands of women who had delivered within the previous 12 months, regardless of the outcome, were included in the study, while husbands who are clinically diagnosed with hearing difficulty were excluded from the study.

### 2.3. Sample Size Determination and Sampling Procedures

The sample size was calculated using a single population proportion formula using 49.3% of the knowledge of pregnancy danger signs during pregnancy, which is taken from a study carried out in northern Ethiopia [15]. The assumption, 95% confidence interval (*z* = 1.96) and 5% margin of error (*d* = 0.05) was considered. The formula for the proportion of single population is as follows: *n* = *Zα*^2^(*P*(1 − *P*)/*D*^2^). n = (Z*α*/2)2.p (1*−*p)/ d2 n = (1.96)2. (0.493) (0.507) / (0.05)2 = 383.9, approximately 384. The final sample size after adding a nonresponse rate of 10% became 422.

**Figure 1:**
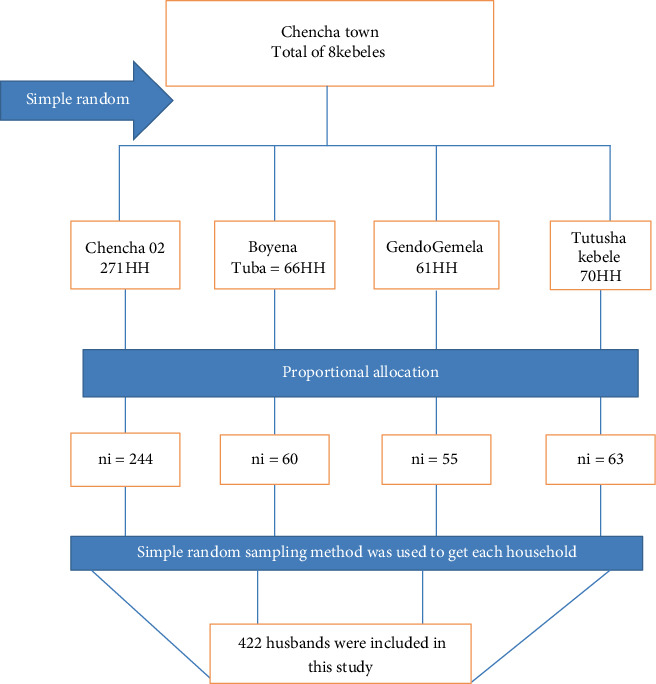
Schematic representation of sampling procedure of husbands' knowledge of pregnancy danger sign and associated factors in Chencha town, southern Ethiopia, 2022.

Chencha town has a total of eight kebeles. Using a simple random sampling method, four kebeles were selected. The total number of eligible households from each kebele was included by proportional allocation. The list of households with husbands of mothers who gave birth within 12 months in the selected kebeles was extracted from the family folder of the health extension workers. Finally, simple random sampling was used to reach and address each study household in kebeles after selecting 422 samples using a computer-generated list of random numbers as shown in Figure 1.

### 2.4. Data Collection Instruments and Procedures

A pretested, interviewer-administered questionnaire was used to collect data from husbands. The data collection tool was prepared after reviewing related literature [8, 15–17]. The questionnaire was translated into Amharic as a local language of the town, then translated to English to maintain consistency. The questionnaire contains four parts: sociodemographic data, social and obstetric part, health and health-related characteristics, and husband knowledge on danger signs during pregnancy.

### 2.5. Study Variables

#### 2.5.1. Dependent Variables

Prevalence of husband's knowledge of Obstetric danger signs during pregnancy.

#### 2.5.2. Independent Variables

The sociodemographic characteristics are as follows: age, religion, ethnicity, education, monthly income, age difference, forms of marriage, and occupation. The social and obstetric-related characteristics are as follows: media exposure, number of children, and ANC follow-up. The health and health-related characteristics are as follows: husband accompaniment during healthcare visit, wife participation in health development army, decision to seek healthcare, and health problem.

### 2.6. Operational Definitions

*Knowledgeable/had good knowledge on the danger sign of pregnancy* refers to those men who responded and scored more than the median value of the 12 questions on the danger signs during pregnancy.

*Not knowledgeable* refers to those men who responded and scored less than or equal to the median value of the 12 questions on the danger signs during pregnancy.

### 2.7. Data Quality Management

During data collection, adequate training and follow-up were provided to data collectors and supervisors. A pretest was performed on husbands outside the study area prior to the actual data collection time, and a correction was carried out. The work of supervisors includes observation of how the data collector administers the questionnaires and random revision of the required number of households. The completeness of the questionnaires was verified by data collectors, supervisors, and researchers on a daily basis. Consequently, any problems encountered were discussed among the data collectors and immediately resolved. Finally, the data was cleaned and organized prior to analysis.

### 2.8. Data Processing and Analysis

After data was collected through mobile phones supporting the Android operating system preinstalled with Open Data Kit (ODK) software, the data were verified for completeness and internal consistency. Then, it was exported to the SPSS Version 25 program for response categorization, coding, and analysis. A descriptive analysis for categorical data was performed, and continuous variables were summarized using means and standard deviation. Data were presented using tables, charts, and text. Binary logistic regression was carried out to determine factors associated with male partner involvement in ANC visits. Factors with *p* values of 0.25 were included in a multivariate logistic regression model to determine predictor variables that were independently associated with the husband's knowledge of pregnancy danger signs. A significant difference was defined as a *p* value less than 0.05.

## 3. Result

### 3.1. Sociodemographic Characteristics of the Study Participants

A total of 400 participants were included in the study, with a response rate of 95% and a nonresponse rate of 5%. The mean age of the study participants was 37.38 ± 8.014 SD and ranged from 20 to 70 years. Almost half of all participants, 53.4% (*n* = 214), were Protestant in religion, and the majority 351 (87.8%) of them had a Gamo ethnic background (see Table 1).

### 3.2. Social and Obstetric-Related Characteristics

From the study, the majority (70.5%) of the participants were exposed to media information on the knowledge of danger signs during pregnancy, while 37% of respondents had three and four children (see Table 2).

### 3.3. Health and Health-Related Characteristics

The following table shows that among 400 husbands, 64.8% of husbands accompanied their partner during healthcare visits and 82.8% of wives participated in health development army (see Table 3).

Among the danger signs during pregnancy, most respondents were aware of blurred vision (87.8%), followed by vaginal bleeding (51.7%), while foul-smelling vaginal discharge was the least acknowledged by husbands as a danger sign during pregnancy (see Table 4).

**Figure 2:**
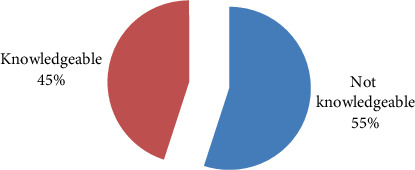
Prevalence of husbands' knowledge on pregnancy danger signs in Chencha town, southern Ethiopia, 2022.

The overall prevalence of husband knowledge on the pregnancy danger sign was 45.5% with 95% CI (41% and 50%) as shown in Figure 2.

### 3.4. Factors Associated With Husband Knowledge of Danger Signs During Pregnancy

Bivariate analysis was performed to select candidate variables for the multivariate analysis by using binary logistic regression. In multivariate analysis, criteria such as a *p* value of 0.25 significance level and biologically plausible variables were used to select and verify the association. Then, 12 variables, including husband's age, husband's education, wife's education, husband's occupation, family income, number of children, access to media information, member of the health development army, previous obstetric complications, and follow-up of antenatal care, were included after checking model fitness.

In multivariate analysis, the following variables were found to have a statistically significant association with husbands' knowledge of danger signs during pregnancy. As mentioned clearly below, multivariate logistic regression analysis showed that there are five variables with a *p* value of less than 0.05. The odds of husbands' knowledge of danger signs during pregnancy were statistically and significantly associated with having secondary education, having higher than secondary education, having media information, having previous complications of pregnancy, and having antenatal care follow-up of greater than or equal to four visits, as shown in Table 5.

## 4. Discussion

The prevalence of good knowledge of the danger sign during pregnancy by husbands is 45.5%, which is consistent with the findings of a study carried out in northeast Ethiopia, south Wollo, Dessie town, and southern Ethiopia, less than the study conducted in Tanzania [15–17]. However, the present study is higher than the research conducted in Nigeria [8]. The disparities could be attributed to the fact that this study is carried out in urban areas and there are differences in sociodemographic status as well as differences in the study period.

This study shows the knowledge of husbands about pregnancy danger signs such as severe abdominal pain 24%, vaginal bleeding 51.7%, severe headache 31.5%, convulsion 27.3%, high-grade fever 26.3%, swollen hands and toes 27.0%, accelerated or reduced fetal heart rate 14.0%, loss of consciousness 12.8%, foul-smelling vaginal discharge 90.8%, sustained vomiting 64.3%, blurred vision 87.8%, and water breaking without labor 19.0%.

Husbands of the wife with an education level of secondary and more than secondary were 4.7 and 3 times more likely to have knowledge about pregnancy danger signs than husbands of the wife without education. A husband of a wife with a previous obstetric complication is 1.8 times more likely to be knowledgeable than a husband of a wife with no history of previous obstetric complication. A husband with access to information media is 1.88 times more likely to be knowledgeable than a husband without access to media information. A husband with antenatal follow-up with his wife for more than four visits is 1.83 times more likely to be knowledgeable than a husband with antenatal follow-up for less than four times.

This study found that husbands of a wife with an educational status of secondary level were 4.7 times more likely to have knowledge than husbands of a wife with no education. In addition to this, husbands of a wife with more than secondary level were 3.1 times more likely to be knowledgeable than husbands of a wife with no education. The result agrees with the research from Ethiopia [15, 18, 19]. The possible reason could be that as women become more educated, they become more empowered and give awareness to their husbands so that husbands are engaged in their wives' health issues and finally become knowledgeable of pregnancy danger signs [20]. However, unlike this, another study found that educational attainment did not have a significant impact on whether respondents had good knowledge of danger signs during pregnancy or not [8]. The possible explanation might be due to the difference in awareness.

The study found that the husband of the wife with a history of previous complications is 1.8 times more likely to be knowledgeable than the husbands of the wife with no history of previous obstetric complications. The finding was consistent with the study done in Ethiopia [15, 21]. This might be explained by the fact that most of the time husbands go to the hospital to support wives during obstetric complications before or after delivery, gaining some clue about the danger signs during pregnancy from healthcare providers.

Husbands of wife with an antenatal care follow-up of greater than or equal to four visits is 1.8 times more likely to be knowledgeable than having an antenatal care follow-up of less than four visits. The reason might be that men with better knowledge of maternal care and pregnancy complications may also be in a better position to be involved in decision-making regarding antenatal care and place of delivery and to advocate for a health facility birth, increasing maternal health service utilization among women, and its vice versa is also true [22]. Another reason might be that if there is a history of antenatal care follow-up, there would be health education or awareness creation by healthcare providers; this also increases husband knowledge regarding pregnancy danger sign.

The study found that a husband with access to media information is 1.88 times more likely to be knowledgeable than husbands with no access to media information about danger signs during pregnancy. The result is in agreement with the research from Ethiopia [23]. The possible reason could be that men with access to media information can gain significant education and more knowledge about danger signs during pregnancy.

## 5. Conclusion and Recommendations of the Study

The proportion of husband knowledge on danger sign during pregnancy was low. The husband's knowledge of danger signs during pregnancy was significantly associated with the wife's educational status, specifically if she had secondary education or higher. It was also linked to having a history of previous obstetric complication, having greater than or equal to four visit antenatal follow-up, and having access to media information. Interventions that address the poor knowledge of danger signs during pregnancy among men, particularly given their role in decision-making within the home, would be valuable. It is recommended to other researchers to conduct another study that shows the knowledge of husbands on danger signs during the postpartum period.

### 5.1. Limitation of the Study

The study was cross-sectional in nature which may make it challenging to determine a cause-and-effect relationship. In addition, this assessment relied on self-reported data, which may introduce recall bias and social desirability bias. The scope of our study was limited to danger signs during pregnancy. However it did not include danger signs during labor and delivery as well as danger signs during postpartum period. The result may not be generalizable due to the limited sample size and the specific geographical location in which the study was conducted. Moreover, this study assessed only husbands' responses; however, both wives and husbands as respondents were under looked.

## Figures and Tables

**Table 1:** Sociodemographic characteristics of study participants (*n* = 400) in Chencha town in southern Ethiopia, 2022.

**Variables**	**Category**	**Frequency**	**Percentage (%)**
Husbands' age (in years)	20–29	71	17.8
	30–39	159	39.8
	≥ 40	170	42.4

Religion	Orthodox	175	43.8
	Muslim	11	2.8
	Protestant	214	53.4

Ethnicity	Gamo	351	87.8
	Amhara	37	9.2
	Oromo	12	3

Education of husbands	No formal education	80	20.0
	Primary	74	18.4
	Secondary	95	23.8
	More than secondary	151	37.8

Family average monthly income	< 4500 ETB	202	50.5
	≥ 4500 ETB	198	49.5

Age difference with wife	≤ 5 years	302	75.5
	> 5 years	98	24.5

Husband occupation	Government employed	268	42.0
	Merchant	139	34.8
	Private worker/farmers	93	23.2

Education of wife	No formal education	113	28.2
	Primary	101	25.3
	Secondary	94	23.5
	More than secondary	92	23.0

Occupation of wife	Government worker	227	56.8
	Private worker/merchant	89	22.2
	Housewife	84	21.0

Forms of marriage	Monogamous	354	88.5
	Polygamous	46	11.5

**Table 2:** Social and obstetric-related characteristics of husbands in Chencha town, southern Ethiopia, 2022.

**Variables**	**Category**	**Frequency**	**Percentage (%)**
Media exposure	No	118	29.5
	Yes	282	70.5

Number of children	1–2 children	147	36.8
	3–4 children	148	37.0
	≥ 5 children	105	26.2

ANC follow-up	< 4 ANC visit	187	46.6
	≥ 4 ANC visit	213	53.3

**Table 3:** Health and health-related characteristics of husbands in Chencha town, southern Ethiopia, 2022.

**Variables**	**Category**	**Frequency**	**Percentage (%)**
Husband accompanying during healthcare visit	No	259	64.8
	Yes	141	35.3

Wife participation in health development army	No	69	17.2
	Yes	331	82.8

Decision to seek healthcare	Husband/respondent	226	56.5
	Pregnant women herself	51	12.8
	Couple	123	30.8

Health problem	No	231	57.8
	Yes	169	42.3

**Table 4:** Prevalence of knowledge of husbands about pregnancy danger signs during pregnancy in Chencha town, southern Ethiopia, 2022.

**Variables**		**Frequency**	**Percentage (%)**
Severe abdominal pain during pregnancy	Yes	96	24.0
	No	304	76.0

Vaginal bleeding	Yes	207	51.7
	No	193	48.3

Severe headache	Yes	126	31.5
	No	274	68.5

Convulsion	Yes	105	27.3
	No	291	72.8

High-grade fever	Yes	105	26.3
	No	295	73.8

Swollen hands and toes	Yes	108	27.0
	No	292	73.0

Accelerated or reduced fetal heart rate	Yes	59	14.0
	No	341	85.3

Loss of consciousness	Yes	51	12.8
	No	349	87.3

Foul-smelling vaginal discharge	Yes	363	90.8
	No	37	9.3

Sustained vomiting	Yes	257	64.3
	No	143	35.8

Blurred vision	Yes	351	87.8
	No	49	12.3

Water break without labor	Yes	76	19.0
	No	324	81.0

**Table 5:** Multivariable regression analysis of husbands' knowledge of danger signs during pregnancy and associated factors among husbands of wives who gave birth in 12 months in the communities of Chencha town, southern Ethiopia, 2022.

**Variables**	**Category**	**Knowledge of pregnancy danger sign**		**95% CI**		**p**
		**Good**	**Poor**	**COR**	**AOR**	
Husband occupation	Government worker	86 (51.2%)	82 (48.8%)	2.098 (1.239–3.552)	1.603 (0.840–3.059)	0.152
	Merchant	65 (46.8%)	74 (53.2%)	1.757 (1.019–3.029)	1.520 (0.819–2.822)	0.185
	Private/farmer	31 (33.3%)	62 (66.7%)	1	1	

Education of wife	No education	31 (27.4%)	82 (72.6%)	1	1	0.134
	Primary education	39 (38.6)	62 (61.4%)	1.664 (0.936–2.958)	1.639 (0.858–3.128)	0.001^∗∗^
	Secondary education	61 (64.9%)	33 (35.1%)	4.890 (2.706–8.836)	4.700 (2.330–9.478)	0.002^∗∗^
	More than secondary	51 (55.4%)	41 (44.6%)	3.290 (1.837–5.894)	3.132 (1.549–6.364)	

Husband education	No education	21 (29.6%)	50 (70.4%)	1	1	
	Primary education	39 (49.4%)	40 (50.6%)	2.321 (1.183–4.555)	1.962 (0.930–4.142)	0.077
	Secondary education	50 (49.5)	51 (50.5)	2.334 (1.229–4.435)	1.315 (0.620–2.789)	0.475
	More than secondary	72 (48.3%)	77 (51.7%)	2.226 (1.219–4.067)	1.363 (0.669–2.774)	0.394

Wife occupation	Government worker	108 (47.6%)	119 (52.4%)	1.403 (0.843–2.335)	1.722 (0.945–3.136)	0.076
	Merchant/private	41 (46.1%)	48 (53.9%)	1.320 (0.721–2.416)	1.151 (0.499–2.655)	0.742
	House wife	33 (39.3%)	51 (60.7%)	1	1	

Place of delivery	Home delivery	18 (31.6%)	39 (68.4%)	1	1	0.769
	Institutional	164 (47.8)	179 (52.2%)	1.985 (1.092–3.607)	1.115 (0.540–2.302)	

Member of health development army	No	42 (60.9%)	27 (39.1%)	1	1	0.284
	Yes	155 (46.8%)	176 (53.2%)	1.370 (0.808–2.326)	1.396 (0.759–2.568)	

Previous obstetric	No	72 (38.3%)	116 (61.7%)	1	1	

Complication	Yes	110 (51.9%)	102 (48.1%)	1.737 (1.166–2.589)	1.796 (1.145–2.817)	0.011^∗^

Access of media information	No	37 (31.4%)	81 (68.6%)	1	1	
	Yes	145 (51.4%)	137 (48.6%)	2.317 (1.472–3.647)	1.881 (1.117–3.166)	0.017^∗^

Antenatal care follow-up	< 4 visit	68 (36.4%)	119 (63.6%)	1	1	
	≥ 4 visit	114 (53.5%)	99 (46.5%)	2.015 (1.349–3.011)	1.839 (1.175–2.880)	0.008^∗∗^

Husband age	20–29	36 (50.7%)	35 (49.3%)	1.469 (0.842–2.564)	1.273 (0.623–2.592)	0.505
	30–39	76 (47.8%)	83 (52.2%)	1.308 (0.846–2.203)	1.116 (0.664–1.876)	0.679
	≥ 40	70 (41.2%)	100 (58.8%)	1	1	

Family income	< 4500 birr	87 (43.1%)	115 (56.9%)	1	1	0.625
	≥ 4500 birr	95 (48.0%)	103 (52.0%)	1.219 (0.822–1.808)	1.122 (0.707–1.780)	

Number of children	1–2	72 (49%)	75 (51%)	1.625 (0.974–2.709)	1.025 (0.529–1986)	0.941
	3–4	71 (48.0%)	77 (52.0%)	1.560 (0.936–2.600)	1.234 (0.680–2.238)	0.489
	≥ 5	39 (37.1%)	66 (62.9%)	1	1	

⁣^∗^*p* < 0.05; ⁣^∗∗^*p* < 0.01.

## Data Availability

The datasets used and/or analyzed during the current study are available from the corresponding author on reasonable request.

## Nomenclature

ANC: antenatal care
AOR: adjusted odds ratio
COR: crude odds ratio
COVID-19: coronavirus disease 2019
ETB: Ethiopian Birr
ODK: Open Data Kit
SDG: Sustainable Development Goal
SPSS: Statistical Package for Social Sciences
WHO: World Health Organization
